# A *MYH7* variant in a five-generation-family with hypertrophic cardiomyopathy

**DOI:** 10.3389/fgene.2024.1306333

**Published:** 2024-02-08

**Authors:** Magda Franke, Tomasz Marcin Książczyk, Marta Dux, Przemysław Chmielewski, Grażyna Truszkowska, Dorota Czapczak, Radosław Pietrzak, Zofia Teresa Bilinska, Urszula Demkow, Bożena Werner

**Affiliations:** ^1^ Department of Pediatric Cardiology and General Pediatrics, Doctoral School, Medical University of Warsaw, Warsaw, Poland; ^2^ Department of Pediatric Cardiology and General Pediatrics, Medical University of Warsaw, Warsaw, Poland; ^3^ Department of Laboratory Diagnostics and Clinical Immunology of Developmental Age, Medical University of Warsaw, Warsaw, Poland; ^4^ Unit for Screening Studies in Inherited Cardiovascular Diseases, Stefan Cardinal Wyszynski National Institute of Cardiology, Warsaw, Poland; ^5^ Department of Medical Biology, Stefan Cardinal Wyszynski National Institute of Cardiology, Warsaw, Poland

**Keywords:** hypertrophic cardiomyopathy, HCM, MYH7, variant, next-generation sequencing

## Abstract

**Background:** Hypertrophic cardiomyopathy (HCM) is a genetic condition with a prevalence of 1:500–1:3 000. Variants in genes encoding sarcomeric proteins are mainly responsible for the disease. *MYH7* gene encoding a myosin heavy chain beta, together with *MYPBC3* gene are the two most commonly affected genes. The clinical presentation of this disease varies widely between individuals. This study aims to report a variant of *MYH7* responsible for HCM in a five-generation family with a history of cardiac problems.

**Methods:** The diagnosis was established according to the European Society of Cardiology HCM criteria based on two-dimensional Doppler echocardiography or cardiovascular magnetic resonance. Genetic analysis was performed using next-generation-sequencing and Sanger method.

**Results:** The medical history of the presented family began with a prenatal diagnosis of HCM in the first child of a family with previously healthy parents. Five generations of the family had a long history of sudden cardiac death and cardiac problems. A NM_000257.4:c.2342T>A (p.Leu781Gln) variant was detected in the MYH7 gene. It was heterozygous in the proband and in all affected individuals in a large family. The variant was present in 10 affected members of the family, and was absent in 7 members. The clinical course of the disease was severe in several members of the family: three family members died of sudden cardiac death, one patient required heart transplantation, three underwent septal myectomy, and three required implantable cardioverter defibrillator (ICD) implantation.

**Conclusion:** Herein, we report a *MYH7* variant responsible for HCM. Familial HCM is inherited primarily in autosomal dominant mode, which is in accordance with our study. However, the presented family showed a broad clinical spectrum of HCM. Out of 10 family members with positive genetic testing 8 had severe presentation of the disease and 2 had a mild phenotype. This suggests that the severity of the disease may depend on other factors, most likely genetic.

## 1 Introduction

According to a recent scientific statement from the American Heart Association, every 1 in 100,000 children will develop cardiomyopathy yearly (Lipshultz et al., 2019). Hypertrophic cardiomyopathy (HCM) is the second most common type of cardiomyopathy and the most common inherited cardiac disease. It is a condition in which left ventricular muscle hypertrophy is present without anomalous afterload conditions, such as hypertension, aortic valve stenosis, or coarctation of the aorta. HCM is a genetically determined condition with a largely autosomal-dominant pattern of inheritance and a complex multigenic background. According to the Human Gene Mutation Database, there are about 60 different genes which harbor several hundred mutations which are responsible for HCM. Genes encoding sarcomeric proteins are most commonly affected (Kaski et al., 2009; Sabater-Molina et al., 2018). *MYH7*, a gene encoding a heavy chain of beta-myosin, and *MYBPC3*, encoding myosin-binding protein C, comprise up to 80% of all genetic causes of HCM (Erdmann et al., 2003; Kaski et al., 2009; Cecchi et al., 2014; Ploski et al., 2016; Ommen et al., 2020).

In this study, we report the case of a family with a history of HCM. We detected a variant in the *MYH7* gene, which segregated with affected members of the described family. We reported this variant for the first time to VarSome and the LOVD database (ID00436735). It has also recently been found in one patient with restrictive cardiomyopathy (RCM) phenotype and published by Szczygiel et al. (Szczygieł et al., 2023).

## 2 Materials and methods

### 2.1 Clinical assessment

The European Society of Cardiology (ESC) HCM criteria were used to establish the diagnosis in the family members. For the proband and her relatives, a two-dimensional Doppler echocardiogram was performed, followed by a standard 12-lead electrocardiogram (ECG) and serum biomarker analyses. HCM was defined as a left ventricular wall thickness greater than 15 mm in adults and a z-score >2 in pediatric family members, as measured using echocardiography or cardiovascular magnetic resonance (CMR). Cardiopulmonary exercise testing was used as an objective method to assess the physical capacity. The CARE guidelines are provided in Supplementary Figure S1.

### 2.2 Genetic assessment

Genomic DNA of the proband and the siblings was extracted from whole blood using ReliaPrep™ Blood gDNA MiniPrep System (Promega, United States of America). DNA purity was checked using a Nanodrop spectrophotometer (Thermo Fisher Scientific, United States). DNA was quantified using a fluorometric method on a Quantus with the QuantiFluor ONE dsDNA System (Promega, United States). Genomic DNA integrity was determined using the Genomic DNA Reagents Analysis Kit on a TapeStation System (Agilent, United States). The library of the proband DNA was prepared using the TruSight Cardio Sequencing Kit (Illumina, United States), and testing was performed using next-generation sequencing (NGS) on the MiSeq System (Illumina). The panel used hybridization probes designed to provide comprehensive coverage of the coding sequences of 174 genes associated with known cardiac diseases, including cardiomyopathies, arrhythmias, and aortopathies. The library preparation procedure consisted of enzymatic DNA fragmentation (tagmentation), amplification of tagmented DNA by adding index adapters, multiplexing of single libraries, double hybridization with probe capture, amplification of the enriched library, and cleaning after every step. The size distribution of the final libraries was determined using a High Sensitivity DNA Analysis Kit on a TapeStation System (Agilent). Final quantification was performed using a fluorometric method. Using Illumina solutions, secondary analysis (alignment and variant calling) was performed on the MiSeq platform. The variants obtained from NGS were analyzed using Variant Interpreter (Illumina, United States), Integrative Genomics Viewer (IGV; Broad Institute), and public genomic databases. The detected variants were verified by Sanger sequencing and analyzed using Mutation Surveyor software (SoftGenetics, United States).

## 3 Results

The patient’s family history began with the diagnosis of HCM in our proband. She was admitted to the Department of Pediatric Cardiology as a 6-day-old newborn of nonconsanguineous, healthy parents. On admission, the patient presented with no symptoms of heart failure.

**FIGURE 1:**
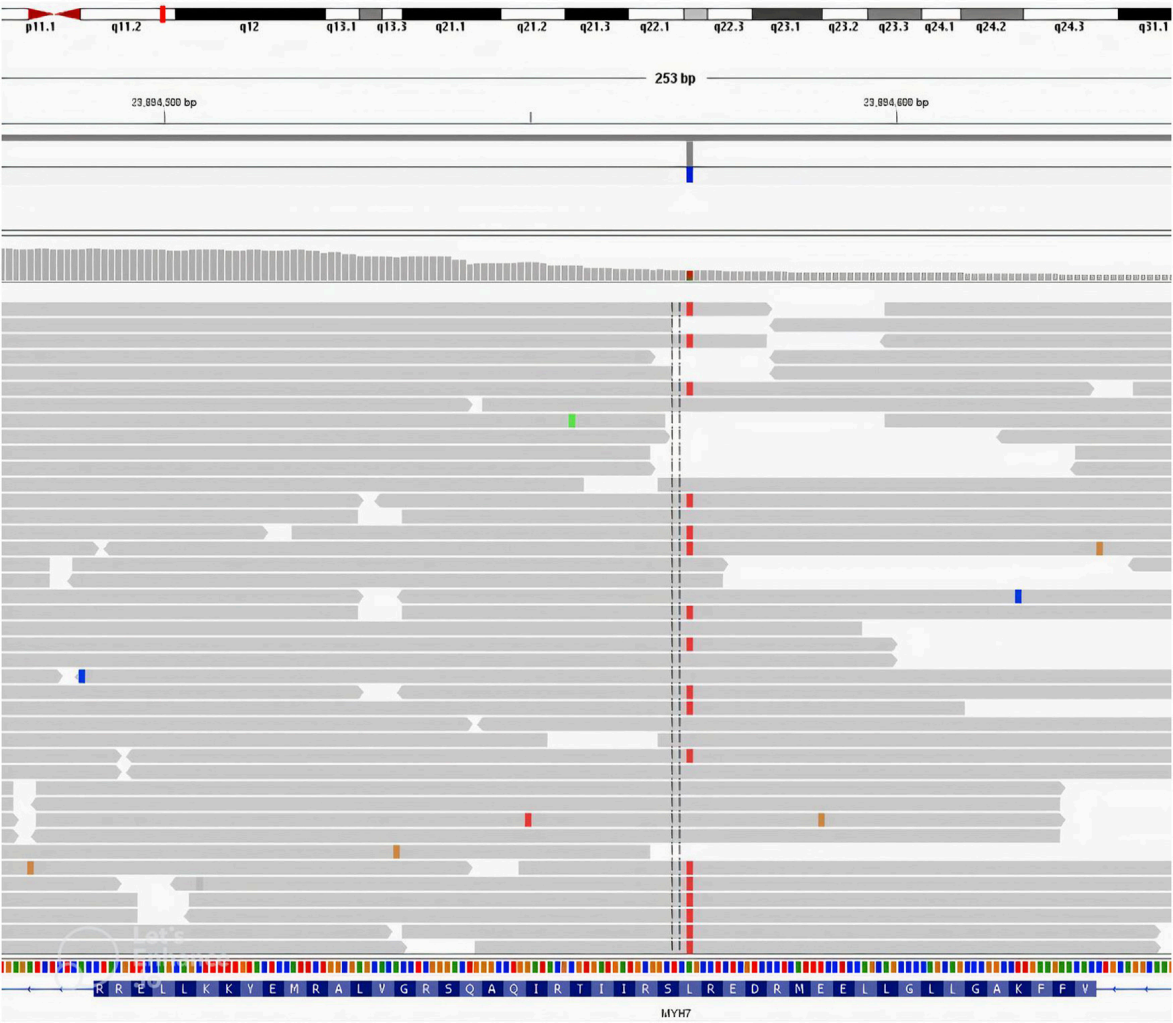
MYH7 (NM_000257.2:c.[2342T>A(p.Leu781Gln) mutation in the proband; NGS result using IGV screen shot.

**FIGURE 2:**
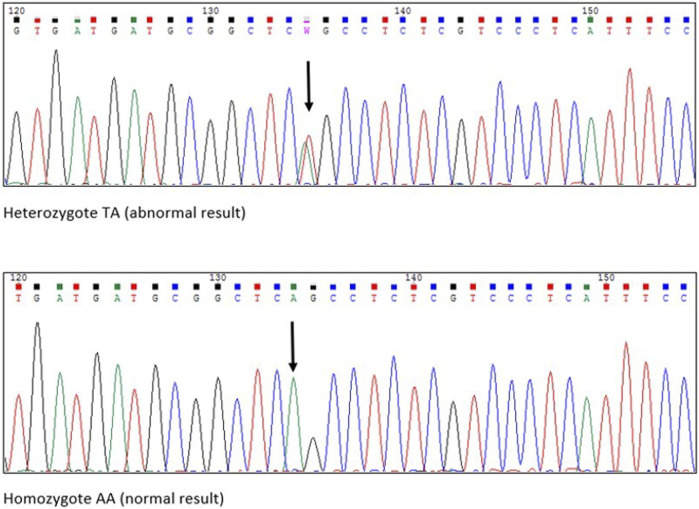
Chromatograms from Sanger sequencing. Black arrows pointing the exact place of amino acid substitiution.

The first postnatal echocardiography of the proband revealed intraventricular septal hypertrophy with an interventricular septum diastolic diameter of 12 mm (Detroit z-score +16, 19). The maximal systolic pressure gradient through the left ventricular outflow tract (LVOT) was 56 mmHg, and systolic anterior motion (SAM) of the mitral valve was observed together with first-degree mitral valve regurgitation. A dynamic left ventricular outflow tract obstruction (LVOTO) was present with a minimal diameter of the LVOT of 2 mm measured in PLAX view during systole. A weight-adjusted dose of propranolol was administered. The patient was followed-up regularly with cardiac biomarkers, ECG, 24-h Holter monitoring, echocardiography, and CMR as soon as the patient was suitable for the procedure without sedation. Additionally, NGS using the TruSight Cardio panel was performed in our proband to verify the clinical findings. Molecular testing revealed the presence of the NM_000257.4:c.2342T>A (p.Leu781Gln) variant in *MYH7* (Figures 1, 2 Supplementary Figure S2). This variant has never been reported before as causative of HCM, and only recently has been found in one patient with RCM (Szczygieł et al., 2023), and is also indicated as a damaging or disease-causing by several bioinformatic tools.

**FIGURE 3:**
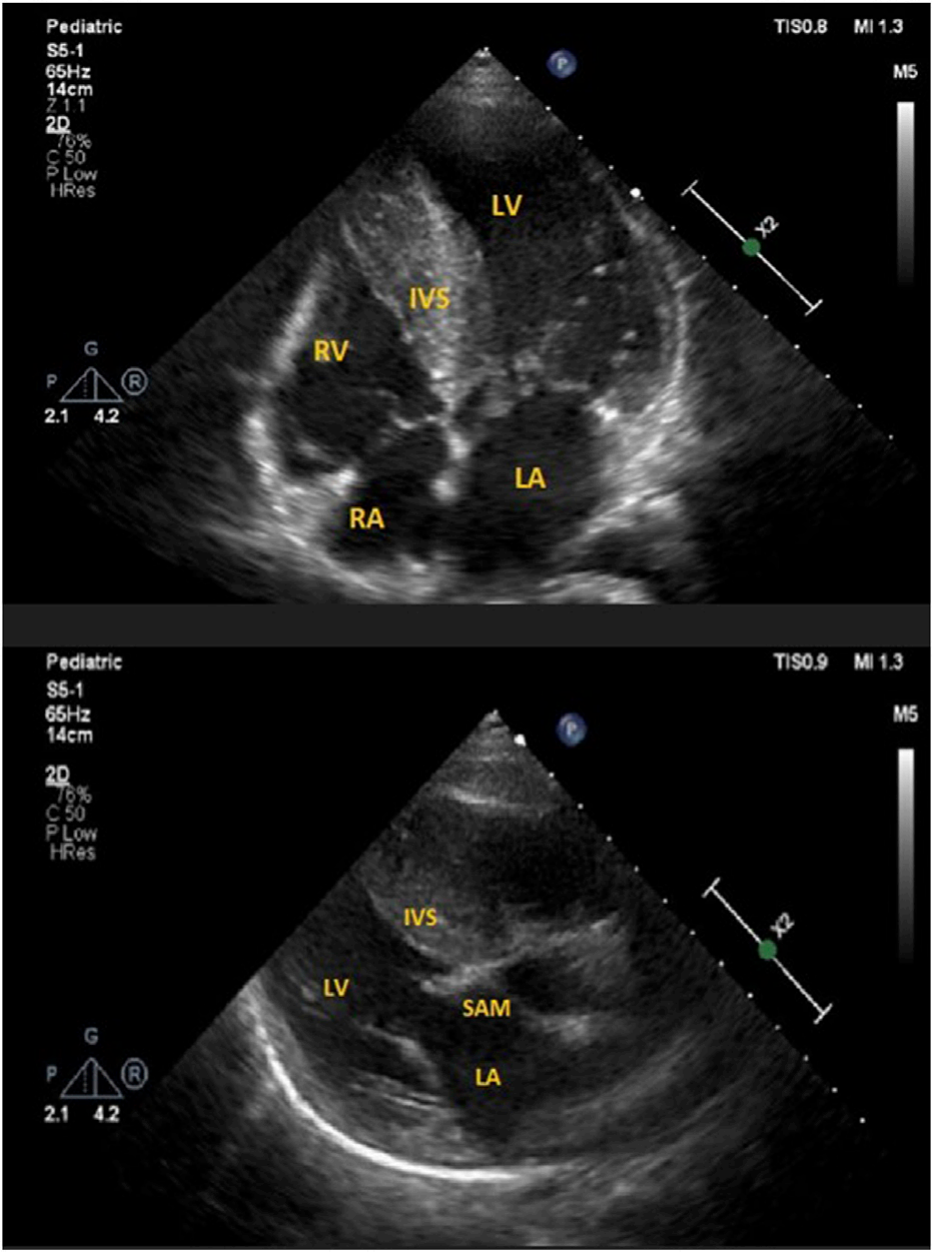
**(A)** ECHO 2-D showing severe interventricular hypertrophy in apical-4 chamber view in the proband. LV, left ventricle; LA, left atrium; RV, right ventricle; RA, right atrium; IVS, intraventricular septum. **(B)** ECHO 2-D showing systolic anterior motion (SAM) of mitral valve in parasternal long axis view in the proband. LV, left ventricle; LA, left atrium; IVS, intraventricular septum.

**FIGURE 4:**
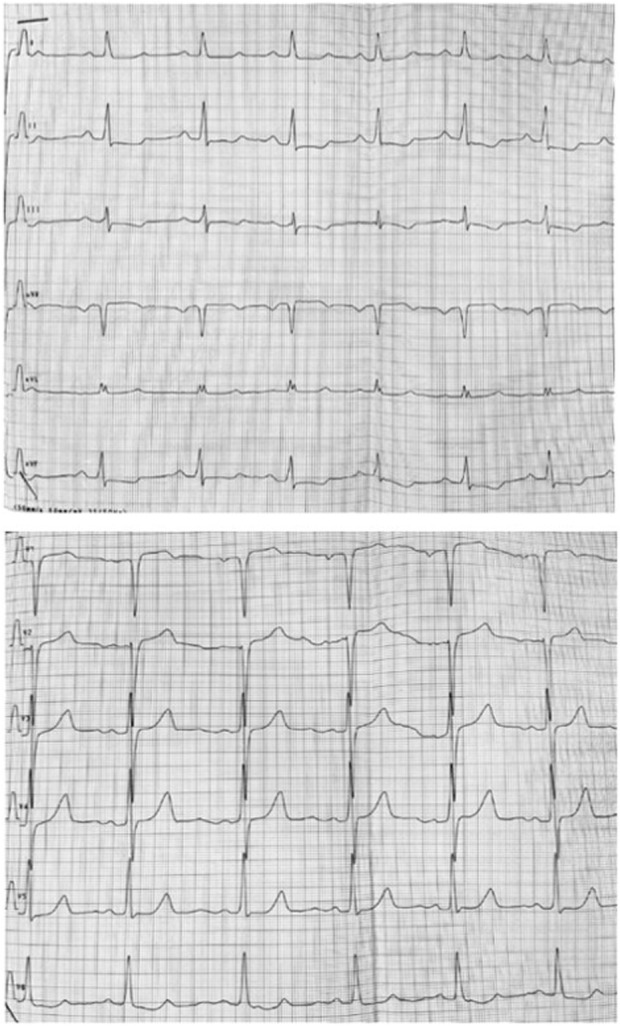
ECG showing hypertrophy of left ventricle and abnormal ST-T segments in multiple leads.

Follow-up visits over 13 years revealed gradual disease progression. Imaging studies showed an increase in the thickening of the intraventricular septum (maximum, 28 mm) and worsening of the LVOT obstruction (maximum, 90 mmHg) (Figures 3A, B). ECG revealed severe left ventricle hypertrophy with abnormal ST-T segments in multiple leads (Figure 4). Nonsustained ventricular tachycardia was detected on Holter ECG. Additionally, CMR revealed late gadolinium enhancement in the lower, middle, and upper IVS segments. Cardiopulmonary exercise tests revealed significantly decreased physical capacity and an abnormal response of systolic blood pressure.

Despite maximal tolerated medical therapy (including disopyramide), the patient remained in NYHA III class with maximal systolic pressure gradient through LVOT over 90 mmHg. Therefore, according to ESC standards, the patient was qualified for surgical myectomy. The procedure significantly reduced the symptoms and improved the quality of life of the patient.

Following the diagnosis in our proband, a cascade of phenotypic and genetic screening was performed in our proband’s first-degree relatives. HCM was found in the proband’s younger brother, father, and paternal grandmother. The mother and sister showed no signs of the condition on ECG or echocardiography. The variant was genetically confirmed in all first-degree relatives who presented with clinical symptoms of HCM; most importantly, the unaffected mother and sister of the index case were found not to carry the variant (asterisked on pedigree).

The father of the proband had a mildly expressed phenotype with asymmetrical hypertrophy of the myocardium, mild SAM of the mitral valve, and maximal systolic pressure gradient in the LVOT of 18 mmHg. The thickness of the intraventricular septum during diastole was 17 mm, that of the inferior segment of the anterior wall was 15 mm, and his ejection fraction continued to be normal. The proband father was qualified for ICD implantation in primary prevention due to high risk of SCD according to ESC guidelines, however due to recent qualification to mavacamten treatment the decision was temporarily postponed. With clinically and genetically confirmed HCM, the grandmother successfully underwent heart transplantation due to severe heart failure (NYHA grade IV).

As mentioned, our proband’s sister remains on regular follow-up and has not shown any signs of HCM. The proband’s brother was prenatally diagnosed with HCM and remained under regular follow-up with a maximal interventricular septum diastolic diameter of 16 mm (Z-score +14.9) and a maximal systolic pressure gradient through the LVOT of 90 mmHg (mean, 42 mmHg). SAM of the mitral valve was observed. No arrhythmias were detected.

**FIGURE 5:**
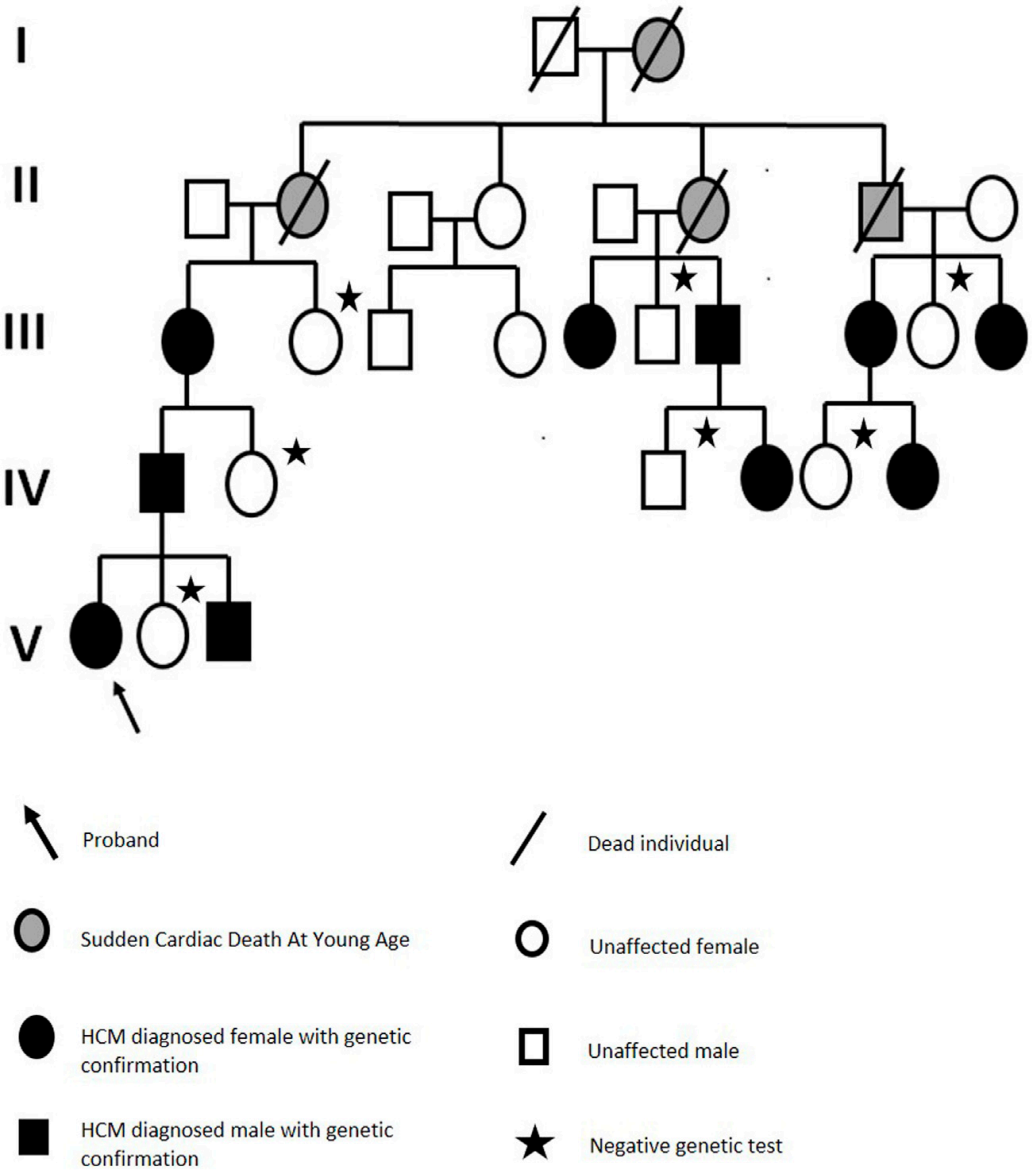
Family pedigree.

Moreover, a thorough family history was collected, including medical reports and imaging test results. This revealed a five-generation history of cardiac disorders. A detailed family history revealed that our proband’s great-great-grandmother was the first diagnosed with a cardiac problem and died of sudden cardiac death at 37. Three of the four children were later diagnosed with a cardiac condition, and two died of sudden cardiac death at less than 40. In total, 14 family members were either diagnosed with HCM or died at a young age owing to sudden cardiac death. Ten individuals harbored this variant. All the phenotypically negative, living relatives were also offered a genetic testing and were found not to be carriers of the reported variant (Figure 5).

To this day, three family members had an ICD implanted with appropriate therapies, one needed a heart transplant, and three had septal myectomies performed. Two other patients, including the proband, qualified for ICD implantation. A 54-year-old female patient has nonobstructive HCM with borderline left ventricular function (LVEF 52%). Her daughter, at 26 years old, has a mild clinical presentation. All family members were regularly examined by cardiologists.

## 4 Discussion

In this study, we report a *MYH7* variant responsible for HCM with LVOTO in a five-generation family. The presented variant has never been reported in the genetic databases, and has only recently been reported to be found in one patient with RCM phenotype in the publication by Szczygieł et al. (Szczygieł et al., 2023). However, based on this isolated finding, there is not enough data to determine whether this variant could be also responsible for the RCM phenotype. This potentially deleterious variant was confirmed in the proband using the NGS TruSight Cardio panel. Importantly, it segregates with affected members of the family. Based on our genetic analysis and the pedigree (Figure 5) we conclude that the mode of inheritance of this variant is autosomal dominant and it shows a complete penetrance.

The family history started with a prenatal diagnosis of HCM in our proband. All possible extrinsic or secondary causes of the disease were excluded (Pedra et al., 2002; Weber et al., 2014). The patient was then transferred to our facility. We performed early clinical and genetic screening of our patient’s first-degree relatives and identified all other affected individuals. Owing to the early recognition of the disease, it was possible to implement early diagnostic and therapeutic management according to the ESC and American Heart Association recommendations (Cecchi et al., 2014; Lipshultz et al., 2019; Ommen et al., 2020). We assessed our patient’s risk of sudden cardiac death using several methods, starting with the risk prediction model for sudden cardiac death in childhood HCM (HCM Risk-Kids) (Kaski et al., 2019) Furthermore, the latest meta-analysis on survival and prognostic factors in HCM published in Scientific Reports (Liu et al., 2017) emphasizes the role of factors, such as family history of sudden death, nonsustained ventricular tachycardia, and LVOTO, as the strongest predictors for all-cause death and sudden cardiac death in patients with HCM. Our patient presented with all the above factors, constituting a strong argument for implementing early and aggressive treatment. CMR reveals late gadolinium enhancement, which increases the risk of ventricular arrhythmias (Brenes et al., 2018). In addition, despite maximally tolerated pharmacological therapy, the patient remained in NYHA class III. Considering all the aforementioned arguments, the patient qualified and underwent a septal myectomy. The patient qualified for subcutaneous ICD implantation.

The affected family members that we present here were diagnosed as carrying a variant located in *MYH7*. According to the ClinVar database, there have been 148 pathogenic and 248 likely pathogenic variants of the *MYH7* gene, of which missense mutations constitute the vast majority. The *MYH7* variant NM_000257.4:c.2342T>A (p.Leu781Gln) described in our study is a single-base exchange resulting in a single amino acid substitution in 781 codons altering Leucine for Glycine (Figures 1, 2). It encodes a normal-length protein with an amino acids (AA) sequence alteration, and 781 codons build the first AA of the IQ domain of the myosin-7 protein. The IQ domain function is strictly associated with calmodulin–calcium-modulated proteins and allows Ca^2+^ signaling, which affects various cellular activities. Overall, the described variant altered the function of the heavy chain of beta-myosin. The detected variant was not registered in dbSNP, HGMD-Public, GnomAD or other available databases containing information on mutations in the human genome. There is no information regarding the variant frequency in the population. The variant is indicated as damaging/disease-causing by all available algorithms. In silico studies predict a pathogenic outcome for this variant (MutationAssessor, SIFT, and REVEL). The VarSome database classifies the variant as pathogenic (with PP3 In-Silico predictors) according to the American College of Medical Genetics and Genomics classification. PP3 means that multiple lines computational evidence support a deleterious effect on the gene or gene product. PM1-6 are a part of pathogenic evidence categories classification and are classified as moderate. Criteria for the variant classification were as follows: PM1 Hot-spot of length 17 amino-acids has 35 missense/in-frame variants (18 pathogenic variants, 17 uncertain variants and no benign), which qualifies as strong pathogenic. UniProt protein MYH7_HUMAN domain ‘IQ’ has 45 missense/in-frame variants (18 pathogenic variants, 26 uncertain variants and 1 benign variant), which qualifies as moderate pathogenic. PP3 MetaRNN = 0.941 is greater than 0.939 ⇒ strong pathogenic. PM5 Alternative variant chr14:23425363 A⇒G (Leu781Pro) is classified Likely Pathogenic by the VarSome community in article 25611685 (confirmed using the germline classifier). PM2 Variant not found in gnomAD genomes, good gnomAD genomes coverage = 31.1.

Two other variants in the same location as MYH7 have been reported (NM_000257.4:c.2342T>C p. Leu781Pro; rs727503259, CM1516000; and NM_000257.4:c.2341C>A p. Leu781Met; rs1348730180, CM1412213) as variants of unknown significance (ClinVar) in patients with hypertrophic cardiomyopathy (HGMD) [PMID:29121657, PMID:28615295].

HCM caused by mutations in genes encoding sarcomeric proteins has an earlier and more severe presentation (Cecchi et al., 2014) placing the patient at a higher risk of cardiovascular events and heart failure than those with negative genetic test results (Olivotto et al., 2008). The phenotypes of family members affected by certain mutations may differ. Although HCM is characterized by high penetrance, some individuals may have milder symptoms or symptoms that occur at an older age. Expressivity may vary because of modifier genes, environmental factors, or lifestyle factors (Lipshultz et al., 2019). Our family demonstrated the heterogeneity of the time of onset of clinical symptoms, as well as their severity.

According to recent publications, there is a tendency to diagnose HCM in older patients (Canepa et al., 2020). This is usually because patients stay asymptomatic until an older age, which may lead to possible complications of non-diagnosed HCM. Early genetic testing is crucial for the identification and diagnosis of these patients. Our case proves that despite early diagnosis, the efficacy of the available pharmacological treatment needs further improvement. According to Carolyn y Ho (Ho et al., 2018), heart failure symptoms and arrhythmias continue to emerge in over 20% of patients. Currently, new therapies are being investigated and could be used in asymptomatic patients to prevent remodeling of the myocardial structure (Repetti et al., 2019). This could conceivably be a future study for patients diagnosed by genetic testing before they develop clinical symptoms of HCM.

## 5 Conclusion

In the present study, we suggest that this *MYH7* missense variant is responsible for the phenotype observed in the affected family members. The patient’s family exhibited a full spectrum of possible symptoms and complications of HCM. Although echocardiography and cardiac magnetic resonance imaging are the two methods approved and used to diagnose cardiomyopathies, this article highlights the importance of genetic evaluation. It can verify a medical diagnosis and help establish a disease-specific prognosis and possible management. Verifying the genetic basis is crucial for predicting the course and severity of the disease. It also discharges patients with negative genetic results from further follow-up. Our study confirms that new variants can still be detected and reported to broaden our genetic knowledge of inherited conditions.

## 6 Limitations of the study

We have reported a 5 generation family with 14 members out of 29 diagnosed with hypertrophic cardiomyopathy or with history of sudden cardiac death at young age. However, it is advisable to seek out other people in whom the same variant was detected in order to verify their clinical course of the disease. The described family shows a great diversity in phenotypical presentation of HCM, and it would be interesting to try to identify factors influencing the different course of the disease.

## Data Availability

The datasets presented in this study can be found in online repositories. The names of the repository/repositories and accession number(s) can be found below: https://databases.lovd.nl/shared/variants/0000933805#00014138, #00436735.
