# Supplementary material for: A MYH7 variant in a five-generation-family with hypertrophic cardiomyopathy
Source: Front Genet. 2024 Feb 8;15:1306333. doi: 10.3389/fgene.2024.1306333 (PMC10883303; doi:10.3389/fgene.2024.1306333)
Supplement: Supplementary file 1 [file DataSheet1.PDF]

CARE Guidelines

1. Title – A MYH7 variant in a five-generation-family with hypertrophic cardiomyopathy

2. Key Words - Hypertrophic cardiomyopathy, HCM, MYH7, Next Generation Sequencing

3. Abstract – (structured or unstructured)

Introduction – The manuscript describes a variant of MYH7 gene which was not previously reported in genetic databases, and it affects 10 family members with a broad spectrum of HCM phenotype. The proband presented a severe course of the disease. She was being observed in our centre since birth and until the age of 12 remained asymptomatic, despite very advanced echocardiography presentation of the disease. Once she started presenting heart failure symptoms, which were objectively confirmed with cardiopulmonary exercise testing, the pharmacological treatment was escalated, and surgical treatment was offered and then performed. The reported MYH7 variant has been found in the proband and then confirmed in other affected members of the family. Nonaffected family members have been found not to carry the variant.

Conclusion – With the presented study we provide evidence for pathogenicity of the reported variant. We would like to emphasize the importance of medical genetic evaluation of the diseases which in modern medicine determines disease-specific prognosis and possible management.

4. Introduction

The presented manuscript is unique as it describes the 5-generation-family affected by HCM with a varied clinical course of the disease, which we believe is caused by the reported MYH7 variant. It is of value as it merges both clinical and genetic perspective.

5. Patient Information

As stated in the Results section of the manuscript: The proband was admitted to the Department of Pediatric Cardiology as a 6-day-old newborn of nonconsanguineous, healthy parents. The diagnosis of the HCM was then established. Following the cascade screening, other members of the family were diagnosed with the condition.

6.Clinical Findings

As stated in the Results section of the manuscript:
At first on admission, the proband presented with no symptoms of heart failure. Follow-up visits over the years revealed gradual disease progression. Cardiopulmonary exercise tests revealed significantly decreased physical capacity and an abnormal response of systolic blood pressure. Despite maximal tolerated medical therapy (including disopyramide), the patient remained in NYHA III class. Therefore, according to ESC standards, the patient was qualified for surgical myectomy. The procedure significantly reduced the symptoms and improved the quality of life of the patient. Detailed clinical findings in other family members are presented in the Results section of the manuscript.
NGS analysis revealed the presence of the reported MYH7 variant in the proband. Following [the HCM] diagnosis in our proband, a cascade of phenotypic and genetic screening was performed in our proband's first-degree relatives. HCM was found in the proband's younger brother, father, and paternal grandmother. The mother and sister showed no signs of the condition on ECG or echocardiography. The variant was genetically confirmed in all first-degree relatives who presented with clinical symptoms of HCM; most importantly, the unaffected family members were found not to carry the variant.

7. Timeline

The proband was first admitted to our facility after birth. She stayed under regular yearly follow-up until the age of 13 when she was qualified to surgical myectomy. Since the diagnosis in the proband other family members have been found to be affected with HCM and referred to specialist care.

8. Diagnostic Assessment

Diagnostic methods were used as stated in the European Society of Cardiology and American Heart Association guidelines on hypertrophic cardiomyopathy management. We stated in the manuscript: The European Society of Cardiology (ESC) HCM criteria were used to establish the diagnosis in the family members. For the proband and her relatives, a two-dimensional Doppler echocardiogram was performed, followed by a standard 12-lead electrocardiogram (ECG) and serum biomarker analyses. HCM was defined as a left ventricular wall thickness greater than 15 mm in adults and a z-score >2 in pediatric family members, as measured using echocardiography or cardiovascular magnetic resonance (CMR). Cardiopulmonary exercise testing was used as an objective method to assess the physical capacity. Genetic testing was performed using the next-generation-sequencing and Sanger method.

9. Therapeutic Intervention

Therapeutic interventions were carried out according to European Society of Cardiology and American Heart Association guidelines. Affected family members received complex pharmacological treatment. Our proband underwent surgical myectomy and was qualified for an ICD implantation as a primary prevention sudden cardiac death. Proband's father has been qualified to treatment with mavacamtem. To this day, three family members had an ICD implanted with appropriate therapies, one needed a heart transplant, and three had septal myectomies performed.

10. Follow-up and Outcomes

As stated in the Results section of the manuscript:
All affected family members are regularly examined by cardiologists. The family members show a very broad spectrum of the severity of the disease. Our proband's great-great-grandmother was the first diagnosed with a cardiac problem and died of sudden cardiac death at 37. Three of the four children were later diagnosed with a cardiac condition, and two died of sudden cardiac death at less than 40. In total, 14 family members were either diagnosed with HCM or died at a young age owing to sudden cardiac death. Ten individuals harbored this variant. All the phenotypically negative, living relatives were also offered a genetic testing and were found not to be carriers of the reported variant.

11. Discussion

The article is of value because of the broad view it gives of the case of familial hypertrophic cardiomyopathy. It presents a variant, which has never previously been reported in any database, and provides evidence for its pathogenicity for HCM in a 5-generation-family. The family members show all possible clinical courses of the disease. However, as we stated in the Limitation section it is advisable to seek out other people in whom the same variant was detected to verify their phenotype and try to identify factors influencing the clinical course of the disease.
Please check the Discussion section of the article for more medical literature.

12. Patient Perspective – N/A.

13. Informed Consent – All family members or their legal guardians described in this report provided consent for publication.
